# Split MutT prevents the mutator phenotype of *mutT*-deficient *Escherichia coli*

**DOI:** 10.1186/s41021-024-00314-8

**Published:** 2024-10-08

**Authors:** Hiroyuki Kamiya

**Affiliations:** Graduate School of Biomedical and Health Sciences, Hiroshima University, 1-2-3 Kasumi, Minami-ku, Hiroshima, 734-8553 Japan

**Keywords:** 8-oxo-7,8-dihydro-dGTP, MutT, Split protein, Nucleotide pool sanitization

## Abstract

**Background:**

The *Escherichia coli* MutT (NudA) protein catalyzes the hydrolysis of an oxidized form of dGTP, 8-oxo-7,8-dihydro-dGTP (8-hydroxy-dGTP), and the spontaneous mutation frequency is elevated in *E. coli* cells deficient in the *mutT* gene.

**Results:**

A split MutT, comprising the N-terminal (residues 1–95) and C-terminal (residues 96–129) peptides, was designed based on the known tertiary structure and linker insertion mutagenesis experiments. The mutator phenotype was complemented when the two peptides were separately expressed in *mutT E. coli* cells.

**Conclusions:**

These results indicated that this split MutT functions as a nucleotide pool sanitization enzyme in vivo.

## Introduction

Nucleoside 5’-triphosphates, DNA and RNA precursors, suffer various types of chemical damage and the lesioned nucleotides cause replicational and transcriptional mutagenesis. DNA repair is expected to minimize the effects of DNA damage on genetic information [1]. Meanwhile, nucleotide pool sanitization, the specific elimination of damaged nucleotides from the nucleotide pool, is universally recognized as a defense against the mutations induced by the damaged nucleotides [2, 3].

The *Escherichia coli* MutT (NudA) protein is a nucleotide pool sanitization enzyme specific for an oxidized form of dGTP, 8-oxo-7,8-dihydro-dGTP (dG^O^TP, 8-hydroxy-dGTP) [4]. The other substrates are the diphosphate derivative 8-oxo-7,8-dihydro-dGDP and the ribo tri-/di-phosphates [5]. The importance of nucleotide pool sanitization is supported by the presence of proteins with similar functions in bacterial and mammalian cells [6–10]. Moreover, the *mutT* deficiency in *E. coli* cells induces a mutator phenotype, and the frequency of A: T->C: G transversions, the signature of dG^O^TP, is elevated in the cells [11]. Thus, the MutT protein is the major system that prevents the mutagenesis induced by oxidized dGTP in bacterial cells [11, 12].

Some proteins can be split into two or more fragments while retaining similar activities to those of the native, uncleaved proteins, and this is called the fragment complementation system. For instance, RNase A digestion by subtilisin cleaves the peptide bond between Ala-20 and Ser-21 to produce the S peptide (residues 1–20) and S protein (residues 21–124). The two peptides reconstitute the noncovalently bound, active complex, RNase S [13]. Another example is *E. coli* β-galactosidase (LacZ). This protein can be separated into two peptides, LacZα and LacZΩ [14]. The β-galactosidase activity becomes functional (positive) when both peptides are present and this phenomenon, α complementation, has been used for cloning genes or gene fragments. Various proteins such as luciferase and enhanced green fluorescent protein have also been split into two parts [15–18] and applied to identify and quantify protein-protein interactions.

In this study, the author examined whether MutT can be split and retain its in vivo activity. First, linker insertion mutagenesis was performed to confirm a possible fragmentation site. Based on the result, the author divided the protein into the N-terminal (residues 1–95, Mu95) and C-terminal (residues 96–129, 96tT) peptides. Plasmid DNAs expressing Mu95 and 96tT, as proteins fused to glutathione-*S*-transferase (GST) and maltose binding protein (MBP), respectively, were introduced into *mutT E. coli* cells. The dual expressions of GST-Mu95 and 96tT-MBP in *E. coli* cells complemented the *mutT* deficiency, as expected. These results indicated that split MutT is functional in vivo.

## Materials and methods

### Materials

The oligodeoxyribonucleotides used for plasmid construction were purchased from Integrated DNA Technologies (Coralville, IA, USA) and Hokkaido System Science (Sapporo, Japan) in purified forms. The pGEX-6P-3 plasmid DNA was from Cytiva (Marlborough, MA, USA). The pET-MBP16b plasmid DNA containing the *E. coli* MBP gene, and the pBAD-MCS plasmid DNA containing the p15A *ori*, the chloramphenicol-resistance gene, the *araBAD* promoter, and the *araC* gene were constructed in our laboratory and will be reported elsewhere.

The pGST_MutT plasmid DNA was constructed in our laboratory by inserting the *E. coli mutT* gene into the pGEX-6P-3 plasmid at the *Bam*HI and *Sal*I sites, to construct the gene encoding the GST-MutT fusion protein. The gene fragments encoding the 1–95 and 96–129 peptides of MutT were separately amplified by PCR, using this plasmid as the template. The two fragments were joined by the linker 5’-d*ATGCAT*TGATAAT*AGATCT*-3’, where the *Nsi*I and *Bgl*II sites are italicized, in the second PCR. This PCR product was inserted into the pGEX-6P-3 vector at the *Bam*HI and *Sal*I sites. The sequence encoding a 30-residue glycine-serine linker, (GGGGS)_6_, 5’-dGGTGGAGGCGGTTCAGGCGGTGGAGGCTCCGGAGGTGGCGGAAGTGGCGGTGGCGGATCAGGTGGAGGTGGCAGCGGAGGCGGAGGTTCC-3’, was inserted at the *Nsi*I and *Bgl*II sites, yielding pGST_MutT_95–96_GlySerLinker. The pGST_MutT_95–96_GlySerLinker(Ala53) was constructed by changing the 53rd codon GAA to GCA. The PCR fragment encoding the N-terminal 95 residues of MutT was inserted into the pGEX-6P-3 plasmid at the *Bam*HI and *Sal*I sites to yield pGST_MutT_1–95. The pGST_MutT_1–95(Ala53) plasmid was constructed by replacing the *mutT(1–95)* gene of pGST_MutT_1–95 with the *mutT(1–95*,*Ala53)* gene, prepared from pGST_MutT_95–96_GlySerLinker(Ala53).

The pBAD-MCS plasmid was digested with *Acc*B1I and *Bst*Z17I, and the chloramphenicol-resistance gene was replaced by the kanamycin-resistance gene, yielding pBAD-MCS-kan^R^. The genes encoding MBP plus the C-terminal His_10_-tag and the terminator sequence were amplified by PCR from pET-MBP16b and inserted into pBAD-MCS-kan^R^ at the *Pst*I site to yield pBAD-MBP. The region corresponding to the 96–129 peptide was amplified by PCR from pGST_MutT_95–96_GlySerLinker and ligated to pBAD-MBP at the *Xba*I and *Pst*I sites, and the Gly-Ser linker was then inserted between the 96tT and MBP genes to yield pBAD-96tT-GSL-MBP. The pTac-MBP_lacI^q^ and pTac-96tT-GSL-MBP_lacI^q^ plasmid DNAs were constructed by replacing the *araC* gene plus the *araBAD* promoter of pBAD-MBP and pBAD-96tT-GSL-MBP, respectively, with the *lacI*^*q*^*-tac* promoter region amplified from pGEX-6P-3.

### *rpoB* assay

KAM0003 *mutT E. coli* cells [19] were transformed by the plasmids containing the wild-type (wt) or mutant *mutT* gene, with and without either the pTac-MBP_lacI^q^ or pTac-96tT-GSL-MBP_lacI^q^ plasmid. The spontaneous *rpoB* mutant frequency was calculated according to the numbers of colonies on the titer and selection (100 µg/mL rifampicin) agar plates, as described previously [19, 20].

## Results and discussion

### Maintained activity of linker-inserted MutT mutant

The *E. coli* MutT protein is the product of the mutator *mutT* gene and catalyzes the hydrolysis of the mutagenic nucleotide dG^O^TP [4]. This protein is a member of the nucleotide hydrolyzing enzymes containing the “Nudix motif”, “MutT signature”, or “phosphohydrolase module” [21, 22]. The motif is GX_5_EX_7_REUXEEXGU (U = hydrophobic amino acid; X = any amino acid) and corresponds to residues 38–60 in MutT. It constitutes part of the phosphate binding portion for the substrate nucleotide. The protein catalyzed the hydrolysis of dG^O^TP/8-oxo-7,8-dihydro-dGDP and their corresponding ribonucleotides, and thus contains the amino acid residues that recognize the base moiety. Residues 23, 28, 35, 77, 78, and 119 reportedly participate in the recognition of the 8-oxo-7,8-dihydroguanine base [23, 24].

**Fig. 1:**
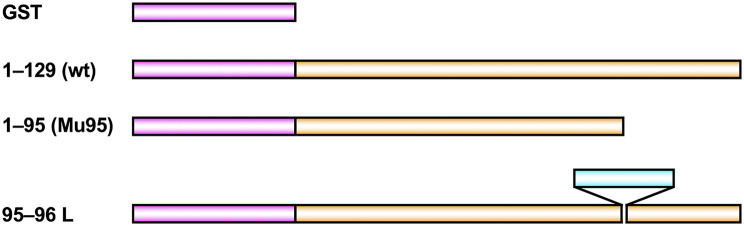
The linker-inserted MutT mutant protein and the control proteins expressed in *E. coli*. The 30-residue glycine-serine linker (GGGGS)_6_ is indicated by the pale blue bar

First, the author hypothesized that the positions for successful fragmentation would tolerate linker insertion mutagenesis. Based on the tertiary structure of MutT, the loop containing residues 95 and 96 was selected [23, 24]. The DNA fragment corresponding to a 30-residue glycine-serine linker, (GGGGS)_6_, was inserted into the *mutT* gene between codons 95 (Trp) and 96 (Gly) (Fig. 1). The mutant was designed to have the amino acid sequence W(95)-M-Q-(GGGGS)_6_-G-S-G(96). This mutant and the wt *mutT* genes were expressed as GST fusion proteins in *E. coli* cells deficient in the *mutT* gene. Spontaneous *rpoB* mutant frequencies were measured by rifampicin resistance.

**Fig. 2:**
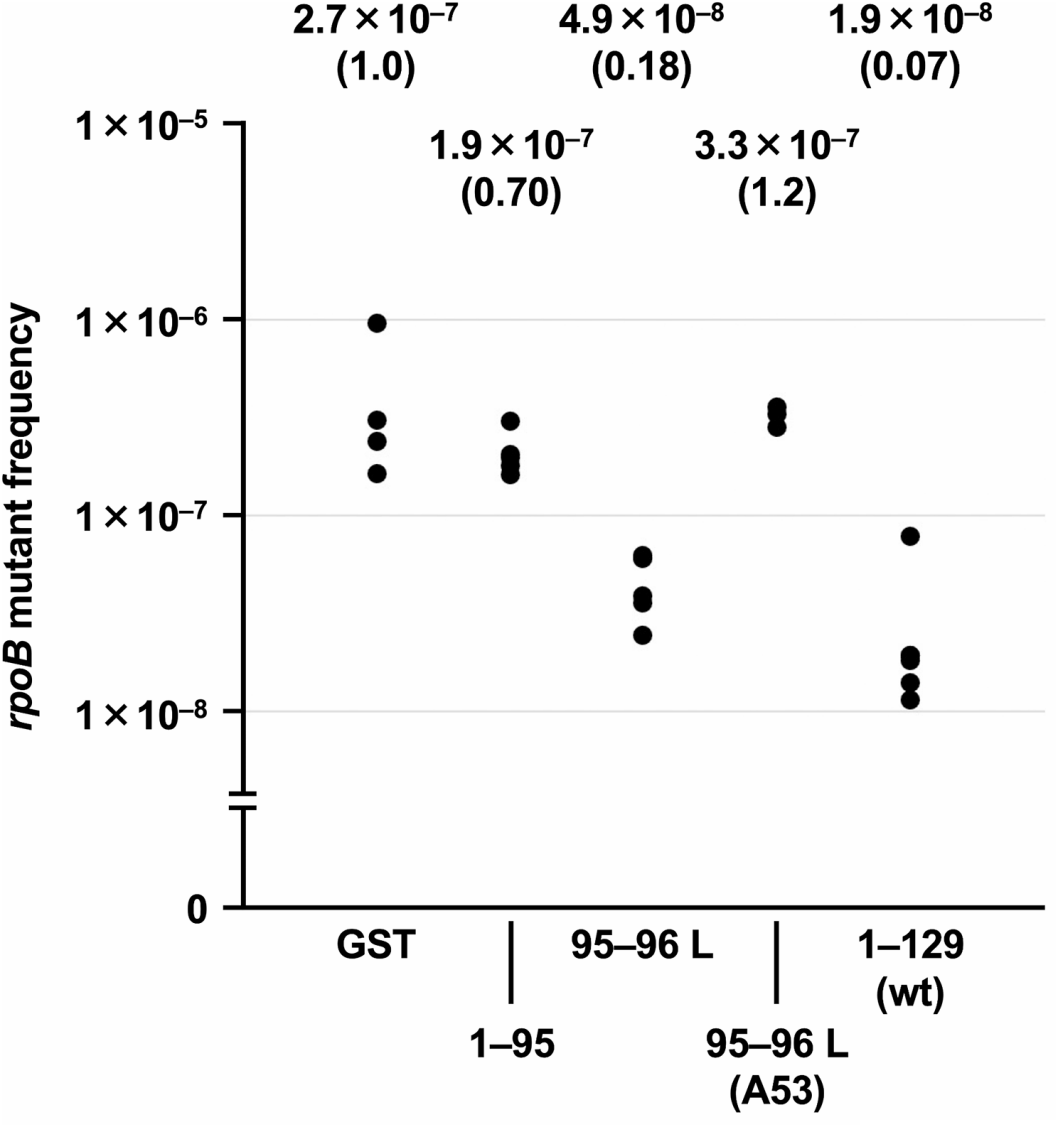
Suppression of spontaneous mutation by expression of the linker-inserted mutant in *mutT* cells. Each dot represents the *rpoB* mutant frequency obtained from a single colony. The median values are shown in the upper part. Their relative values are also shown in parentheses. The 95% confidence interval values are 1.1 × 10^–7^ – 9.3 × 10^–7^, 1.5 × 10^–7^ – 2.6 × 10^–7^, 3.1 × 10^–8^ – 6.4 × 10^–8^, 2.5 × 10^–7^ – 4.1 × 10^–7^, and 1.1 × 10^–8^ – 4.0 × 10^–8^ for GST, 1–95, 95–96 L, 95–96 L (A53), and 1–129 (wt), respectively

As shown in Fig. 2, the truncated mutant (1–95) did not complement the *mutT* deficiency. Meanwhile, the *rpoB* mutant frequency was lower when the linker-inserted mutant 95–96 L was expressed in the *mutT* cells. These results indicated that the inserted glycine-serine linker did not disrupt the catalytic activity of the MutT protein. Moreover, the author substituted Glu-53, an essential amino acid for catalysis, to Ala-53 in the 95–96 L MutT protein [24–26], and observed that the suppressive activity of 95–96 L MutT was lost by this substitution (Fig. 2). Thus, the author decided to divide MutT between Trp-95 and Gly-96.

## Mutation suppression by expression of split MutT

**Fig. 3:**
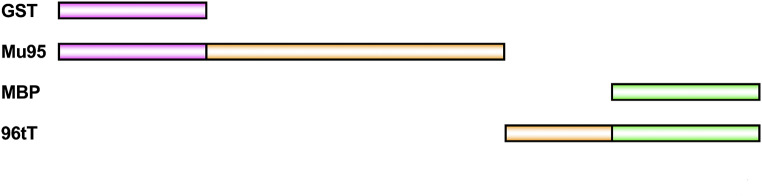
The proteins expressed in *E. coli* to evaluate the activity of split MutT. Mu95, GST-Mu95; 96tT, 96tT-MBP

Next, the anti-mutator function of split MutT was examined. The N-95 residue (1–95) fragment was expressed as a GST fusion protein, GST-Mu95, as described above. The C-34 residue (96–129) fragment was expressed as an MBP fusion protein, 96tT-MBP (Fig. 3).

**Fig. 4:**
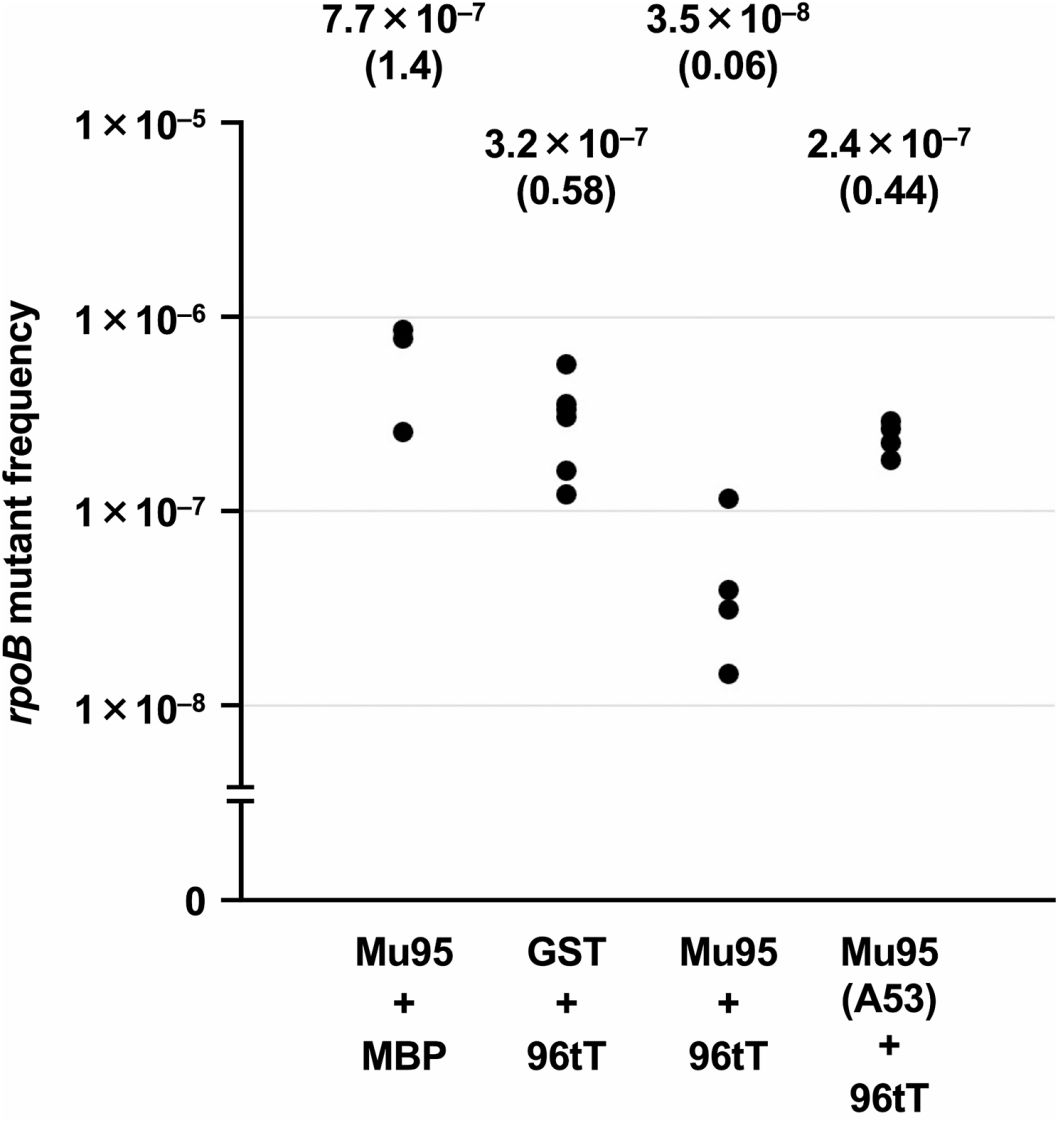
Suppression of spontaneous mutation by split MutT expression in *mutT* cells. Each dot represents the *rpoB* mutant frequency obtained from a single colony. The median values are shown in the upper part. Their relative values, calculated using the mean of *rpoB* mutant frequencies of Mu95 + MBP and GST + 96tT as 1.0, are also shown in parentheses. The 95% confidence interval values are 1.4 × 10^–7^ – 2.1 × 10^–6^, 1.6 × 10^–7^ – 4.6 × 10^–7^, 1.1 × 10^–8^ – 1.2 × 10^–7^, and 1.8 × 10^–7^ – 3.1 × 10^–7^ for Mu95 + MBP, GST + 96tT, Mu95 + 96tT, and Mu95 (A53) + 96tT, respectively. Mu95, GST-Mu95; 96tT, 96tT-MBP

The author co-introduced two plasmid DNAs expressing GST-Mu95 and 96tT-MBP into *mutT*-deficient *E. coli* cells, and measured the spontaneous *rpoB* mutant frequencies. As shown in Fig. 4, the co-expression of GST-Mu95 and 96tT-MBP successfully suppressed the appearance of *rpoB* mutants. When the GST-Mu95 protein with the Ala-53 residue was expressed, the suppressive effect was lost. Thus, the separate expressions of Mu95 and 96tT result in the active complex formation and the hydrolysis of the substrates in *E. coli* cells.

## Association of split fragments

In this study, MutT was split into Mu95 and 96tT, and the two fragments were separately expressed as proteins fused to GST and MBP, respectively, in *E. coli*. The dual expression complemented the *mutT* deficiency (Fig. 4), suggesting that the two parts associated and degraded dG^O^TP in the cells. When Glu-53, a catalytically important amino acid residue in Mu95, was mutated, the complementarity was lost. Thus, the two fragments seemed to have the ability to catalyze the hydrolysis of dG^O^TP in the complex.

The FK506 binding protein (FKBP) and the FKBP-rapamycin binding (FRB) domain of the mammalian target of rapamycin (mTOR) form a heterodimer in the presence of rapamycin, an antifungal antibiotic [27, 28]. Likewise, the Mu95 and 96tT peptides might interact depending on dG^O^TP. Analyses of the binary complex of MutT plus the nucleotide indicated that amino acids 23, 28, 35, 77, 78, and 119 are important for the recognition of the 8-oxo-7,8-dihydroguanine base [23, 24]. The Mu95 and 96tT fragments contain residues 23–78 and 119, respectively, and O6 and N7-H of the base moiety are recognized by Asn-119. Since N7-H is present in 8-oxo-7,8-dihydroguanine but not in guanine, this residue could be crucial for dG^O^TP binding. Thus, the association of split fragments is suggested to be promoted by the oxidized nucleotide. This speculation is supported by our recent observation of split MutT [29]. The interaction of Ash (assembly helper tag)-Mu95 with the Ala-53 residue and hAG (Azami Green)-96tT allowed the visualization of intracellular dG^O^TP by fluorescent foci formation: the fluorescent foci are highly increased when dG^O^TP, but not dGTP, is introduced into human cells. Taken together, the association of Mu95 and 96tT probably occurs in the presence of dG^O^TP or other substrates.

## Data Availability

Data will be made available on request.

## Abbreviations

dG^O^TP: 8-oxo-7,8-dihydro-dGTP
GST: glutathione-*S*-transferase
MBP: maltose binding protein
wt: wild-type
